# Predictive model for identifying mild cognitive impairment in patients with type 2 diabetes mellitus: A CHAID decision tree analysis

**DOI:** 10.1002/brb3.3456

**Published:** 2024-03-07

**Authors:** Rehanguli Maimaitituerxun, Wenhang Chen, Jingsha Xiang, Yu Xie, Fang Xiao, Xin Yin Wu, Letao Chen, Jianzhou Yang, Aizhong Liu, Wenjie Dai

**Affiliations:** ^1^ Department of Epidemiology and Health Statistics, Xiangya School of Public Health Central South University Changsha Hunan China; ^2^ Department of Nephrology Xiangya Hospital, Central South University Changsha Hunan China; ^3^ Department of Human Resources Jinan Central Hospital Affiliated to Shandong First Medical University Jinan Shandong China; ^4^ Department of Toxicology, Xiangya School of Public Health Central South University Changsha Hunan China; ^5^ Infection Control Center Xiangya Hospital, Central South University Changsha Hunan China; ^6^ Department of Preventive Medicine Changzhi Medical College Changzhi Shanxi China

**Keywords:** decision tree, mild cognitive impairment, predictor variable, type 2 diabetes mellitus

## Abstract

**Background:**

As the population ages, mild cognitive impairment (MCI) and type 2 diabetes mellitus (T2DM) become common conditions that often coexist. Evidence has shown that MCI could lead to reduced treatment compliance, medication management, and self‐care ability in T2DM patients. Therefore, early identification of those with increased risk of MCI is crucial from a preventive perspective. Given the growing utilization of decision trees in prediction of health‐related outcomes, this study aimed to identify MCI in T2DM patients using the decision tree approach.

**Methods:**

This hospital‐based case–control study was performed in the Endocrinology Department of Xiangya Hospital affiliated to Central South University between March 2021 and December 2022. MCI was defined based on the Petersen criteria. Demographic characteristics, lifestyle factors, and T2DM‐related information were collected. The study sample was randomly divided into the training and validation sets in a 7:3 ratio. Univariate and multivariate analyses were performed, and a decision tree model was established using the chi‐square automatic interaction detection (CHAID) algorithm to identify key predictor variables associated with MCI. The area under the curve (AUC) value was used to evaluate the performance of the established decision tree model, and the performance of multivariate regression model was also evaluated for comparison.

**Results:**

A total of 1001 participants (705 in the training set and 296 in the validation set) were included in this study. The mean age of participants in the training and validation sets was 60.2  ±  10.3 and 60.4  ±  9.5 years, respectively. There were no significant differences in the characteristics between the training and validation sets (*p* > .05). The CHAID decision tree analysis identified six key predictor variables associated with MCI, including age, educational level, household income, regular physical activity, diabetic nephropathy, and diabetic retinopathy. The established decision tree model had 15 nodes composed of 4 layers, and age is the most significant predictor variable. It performed well (AUC = .75 [95% confidence interval (CI): .71–.78] and .67 [95% CI: .61–.74] in the training and validation sets, respectively), was internally validated, and had comparable predictive value compared to the multivariate logistic regression model (AUC = .76 [95% CI: .72–.80] and .69 [95% CI: .62–.75] in the training and validation sets, respectively).

**Conclusion:**

The established decision tree model based on age, educational level, household income, regular physical activity, diabetic nephropathy, and diabetic retinopathy performed well with comparable predictive value compared to the multivariate logistic regression model and was internally validated. Due to its superior classification accuracy and simple presentation as well as interpretation of collected data, the decision tree model is more recommended for the prediction of MCI in T2DM patients in clinical practice.

## Graphical abstract

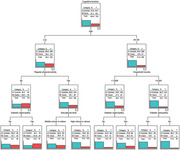

## 1 INTRODUCTION

Mild cognitive impairment (MCI) and type 2 diabetes mellitus (T2DM) are highly prevalent and often coexist in older adults (Srikanth et al., 2020). MCI is a transitional stage between normal ageing and dementia that was characterized by cognitive dysfunction with minimal impairment in instrumental activities of daily living (Petersen, 2004; Petersen et al., 1999). Accumulated evidence has consistently shown that the presence of MCI in the general population was associated with increased risk of dementia (Davis et al., 2018; Mitchell & Shiri‐Feshki, 2009; Zhang et al., 2021). In addition to that, MCI in T2DM could lead to reduced treatment compliance, medication management, and self‐care ability (Kim & Fritschi, 2021; Verma et al., 2021), which may be explained by the decrements in working memory, learning, executive function, and processing speed observed in MCI (Christman et al., 2010; Santos et al., 2018), though the function of daily activities is essentially preserved. Therefore, the management of MCI in T2DM patients is crucial, and early identification of those with MCI is a starting point.

The decision tree is one of the most commonly used machine learning models in a wide range of medical situations requiring decision‐making that provides high classification accuracy with a simple representation of gathered knowledge (Bae, 2014; Podgorelec et al., 2002). Compared to other machine learning models, the decision tree has the following advantages: (1) it can be visualized and is simple to understand and interpret in clinical practice; (2) it provides a remarkably transparent decision‐making process, allowing deep exploration of features; and (3) due to its high transparency, the decision‐making process can be easily validated by an expert that greatly enhances its utility in situations containing high uncertainty (Amendolara et al., 2023; Bae, 2014; Plante et al., 1986; Podgorelec et al., 2002; Ting Sim et al., 2023). A growing body of literature has demonstrated the effectiveness of decision trees in predicting the occurrence as well as the prognosis of health‐related outcomes (Toyoda et al., 2023; Yang et al., 2021; Zhou et al., 2023), and some studies directly compared decision tree model with common machine learning models to solve prediction problems (Hu et al., 2022; Langenberger et al., 2023). Previous work on comparison of logistic regression and decision tree models found comparable predictive values (Wentzlof et al., 2019; Zhang et al., 2022). However, as the interpretation and understanding of logistic regression model are more difficult than that of decision tree model, especially for those without experience with this particular model type, decision tree model is more recommended in clinical practice (Fu et al., 2022). In the diabetic population, few studies to date have used the decision tree models to predict diabetic comorbidities or complications (Kasbekar et al., 2017; Rinkel et al., 2020; Zhou et al., 2022), and to our knowledge, there is no study on prediction of MCI in patients with T2DM using the decision tree model. Given the growing utilization of decision trees in prediction of health‐related outcomes and the negative effects of MCI on the prognosis of T2DM patients, this study aimed to identify MCI in T2DM patients using the decision tree approach to help better identify MCI.

## 2 METHODS

This study adhered to the principles of the Declaration of Helsinki. The protocol of this study was reviewed and approved by the Ethics Committee of the Xiangya School of Public Health, Central South University. Before enrollment, a written informed consent was obtained from each participant.

### 2.1 Study design and setting

This was a hospital‐based case–control study that was performed in the Endocrinology Department of Xiangya Hospital affiliated to Central South University between March 2021 and December 2022, which is a Grade III general hospital and located in the capital city of Hunan Province in China with patients across the country.

### 2.2 Participants

The inclusion criteria for the participants were as follows: (1) a clinical diagnosis of T2DM by the established Chinese Guideline (Chinese Diabetes Society, 2021); (2) hospitalized due to T2DM; (3) aged ≥40 years; and (4) voluntarily participated in this study and signed informed consent. The exclusion criteria for the participants were as follows: (1) repeated hospitalizations during the study period; (2) those with dementia; and (3) those could not speak or understand Mandarin. Eligible participants who met the diagnostic criteria for MCI were considered cases, whereas those unfulfilled were considered controls with normal cognitive function.

### 2.3 Outcome of interest

The outcome of this study is MCI, and it was defined based on the Petersen criteria by the physicians (Petersen, 2004): (1) chief complaint of memory loss confirmed by families or caregivers; (2) objective cognitive impairment measured by the mini–mental state examination score (≤19 for illiterate individuals, ≤22 for individuals with elementary school education, and ≤26 for individuals with middle school education or above) (Fan et al., 2021; Li et al., 2016); (3) essentially preserved function of daily activities; and (4) absence of dementia.

### 2.4 Data collection

Demographic characteristics and lifestyle factors were obtained retrospectively through face‐to‐face interviews by well‐qualified investigators. T2DM‐related information was extracted from medical records. All investigators underwent unified training and were blinded to the cognitive status of the participants.

### 2.5 Predictor variables

The demographic characteristics included age (40–59 vs. ≥60), sex (male vs. female), marital status (married vs. unmarried), educational level (middle school or below vs. high school or above), household income (≤5000 RMB vs. >5000 RMB), location of residence (urban vs. rural), primary caregiver (self‐care vs. others), and current work status (employed vs. unemployed).

Lifestyle factors included information on smoking, drinking, and physical activity, which were all regarded as dichotomous variables (current smoker [Yes vs. No], current drinker [Yes vs. No], and regular physical activity [Yes vs. No]). Specifically, a current smoker was defined as one who smoked at least one cigarette per day in the past month; a current drinker was defined as one who consumed at least one alcohol per day in the past month; and regular physical activity was defined as performing at least one activity such as walking, square dancing, or cycling for at least 150 min per week in the past month (Umpierre et al., 2011; World Health Organization [WHO], 2020).

T2DM‐related factors included information on body mass index (<24 vs. ≥24 kg/m^2^), duration of diabetes (<10 years vs. ≥10 years), family history of diabetes (Yes vs. No), glycated hemoglobin A1c (≤7% vs. >7%), diabetic comorbidities (stroke [Yes vs. No], hypertension [Yes vs. No], coronary heart disease [Yes vs. No], and fatty liver [Yes vs. No]), and diabetic complications (diabetic nephropathy [Yes vs. No], diabetic retinopathy [Yes vs. No], and diabetic foot [Yes vs. No]).

### 2.6 Statistical analyses

The study sample was randomly divided into the training and validation sets in a 7:3 ratio. The training set was used to develop the decision tree model and the validation set was used to validate the decision tree model internally. Based on the fact that excluding all observations with missing values could induce substantial bias as well as lack of efficiency, missing data were filled using random forest interpolation (Salgado, et al., 2016; Fox‐Wasylyshyn & El‐Masri, 2005).

Continuous variables were reported as mean ± standard deviation or median and interquartile range as appropriate, and categorical variables were reported as frequency (*n*) and proportion (%). Chi‐square test or Fisher's exact test was used to compare the categorical predictor variables in the training set as appropriate, and predictor variables that differed significantly were entered into the multivariate logistic regression model.

The chi‐square automatic interaction detection (CHAID) algorithm, whose main purpose was to identify key factors related to the outcomes of interest, was used to develop the decision tree model (Kass, 1980). In this algorithm, homogenous groups could be constructed by any possible combination of the known values of a predictor variable. The number of predictor variables for creating the decision tree model depends on the values of chi‐square test and whether the differences are statistically significant or not. There are three types of nodes in decision tree: (1) a root node, representing a choice that will induce the subdivision of all records into two or more mutually exclusive subsets; (2) internal nodes, representing one of the possible choices that are available at that point in the tree structure; and (3) leaf nodes, representing the final result of a combination of decisions (Song & Lu, 2015). The node containing all the cases is considered the root node in the CHAID algorithm, and the predictor variable with the largest chi‐square value divides the entire sample into at least two subgroups, which are subsequently split by the next most significant predictor variable. The analysis continues in this stepwise way to choose the next most significant predictor variable until there are no more significant predictor variables.

The performance of the decision tree model and multivariate logistic regression model was evaluated by the receiver operating characteristic (ROC) curves and the area under the curve (AUC). All statistical analyses were two‐sided and performed using the IBM SPSS software (version 26.0) or R software (version 4.2.1; https://www.r‐project.org/). A *p* value of <.05 was considered statistically significant.

## 3 RESULTS

### 3.1 Characteristics of the study participants

A total of 1001 participants (274 cases and 727 controls) were included in this study, 705 (191 cases and 514 controls) and 296 (83 cases and 213 controls) of which were randomly assigned into the training and validation sets, respectively. The characteristics of the study participants are shown in Table 1. The mean age of participants in the training and validation sets was 60.2  ±  10.3 and 60.4  ±  9.5 years, respectively, and there were 432 (61.3%) and 181 (61.1%) male participants in the training and validation sets, respectively. There were no significant differences in the characteristics between the training and validation sets (*p* > .05).

**TABLE 1:** Characteristics of the study participants.

Variables	Category	Total sample (*n* = 1001, %)	Training set (*n* = 705, %)	Validation set (*n* = 296, %)	*p* Value
Age (year)	40–59	520 (51.9)	365 (51.8)	155 (52.4)	
	≥60	481 (48.1)	340 (48.2)	141 (47.6)	.864
Sex	Male	613 (61.2)	432 (61.3)	181 (61.1)	
	Female	388 (38.8)	273 (38.7)	115 (38.9)	.970
Marital status	Married	924 (92.3)	649 (92.1)	275 (92.9)	
	Unmarried	77 (7.7)	56 (7.9)	21 (7.1)	.650
Educational level	Middle school or below	542 (54.1)	387 (54.9)	155 (52.4)	
	High school or above	459 (45.9)	318 (45.1)	141 (47.6)	.464
Household income (RMB)	≤5000	638 (63.7)	450 (63.8)	188 (63.5)	
	>5000	363 (36.3)	225 (36.2)	108 (36.5)	.924
Location of residence	Urban	595(59.4)	474 (67.2)	198 (66.9)	
	Rural	406 (40.6)	231 (32.8)	98 (33.1)	.916
Primary caregiver	Self‐care	438 (43.8)	314 (44.5)	124 (41.9)	
	Others	563 (56.3)	391 (55.5)	172 (58.1)	.441
Current work status	Employed	326 (32.6)	229 (32.5)	97 (32.8)	
	Unemployed	675 (67.4)	476 (67.5)	199 (67.2)	.929
Current smoker	No	561 (56)	565 (80.1)	235 (79.4)	
	Yes	440 (44)	140 (19.9)	61 (20.6)	.878
Current drinker	No	626 (62.5)	611 (86.7)	263 (88.9)	
	Yes	375 (37.5)	94 (13.3)	33 (11.1)	.343
Regular physical activity	No	393 (39.3)	274 (38.9)	119 (40.2)	
	Yes	608 (60.7)	431 (61.9)	177 (59.8)	.693
BMI (kg/m^2^)	<24	524 (52.3)	362 (51.3)	162 (54.7)	
	≥24	477 (47.7)	343 (48.7)	134 (45.3)	.328
HbA1c (%)^a^	≤7	323 (32.3)	218 (30.9)	105 (35.5)	
	>7	678 (67.7)	487 (69.1)	191 (64.5)	.160
Duration of diabetes (year)	<10	462 (46.2)	322 (45.7)	140 (47.3)	
	≥10	539 (53.8)	383 (54.3)	156 (52.7)	.640
Family history of diabetes	No	563 (56.2)	386 (54.8)	177 (59.8)	
	Yes	438 (43.8)	319 (45.2)	119 (40.2)	.142
Stroke	No	876 (87.5)	619 (87.8)	257 (86.8)	
	Yes	125 (12.5)	86 (12.2)	39 (13.2)	.670
Hypertension	No	363 (36.3)	252 (35.7)	111 (37.5)	
	Yes	638 (63.7)	453 (64.3)	185 (62.5)	.600
Coronary heart disease	No	786 (78.5)	547 (77.6)	239 (80.7)	
	Yes	215 (21.5)	158 (22.4)	57 (19.3)	.267
Fatty liver	No	739 (73.8)	517 (73.3)	222 (75.0)	
	Yes	262 (26.2)	188 (26.7)	74 (25.0)	.584
Diabetic nephropathy	No	493 (49.3)	347 (49.2)	146 (49.3)	
	Yes	508 (50.7)	358 (50.8)	150 (50.7)	.976
Diabetic retinopathy	No	613 (6.2)	434 (61.6)	179 (60.5)	
	Yes	388 (38.8)	271 (38.4)	117 (39.5)	.747
Diabetic foot	No	922 (92.1)	647 (91.8)	275 (92.9)	
	Yes	79 (7.9)	58 (8.2)	21 (7.1)	.544

Abbreviations: BMI, body mass index; HbA1c, glycated hemoglobin A1c; RMB, renminbi.

^a^ indicated there were 45 missing values of this variable, which was filled using the random forest interpolation.

### 3.2 Univariate analyses of factors associated with MCI in the training set

The univariate analyses of factors associated with MCI in the training set are shown in Table 2. Age, sex, marital status, educational level, household income, location of residence, primary caregiver, current work status, current smoker, current drinker, regular physical activity, duration of diabetes, stroke, hypertension, coronary heart disease, fatty liver, diabetic nephropathy, diabetic retinopathy, and diabetic foot differed significantly between the cases and controls in the training set (*p* < .05).

**TABLE 2:** Univariate analyses of factors associated with MCI in the training set.

Variables	Category	Cases (*n* = 191, %)	Controls (*n* = 514, %)	*χ^2^* Value	*p* Value
Age (year)	40–59	57 (29.8)	308 (59.9)		
	≥60	134 (70.2)	206 (40.1)	50.46	<.001
Sex	Male	105 (55.0)	327 (63.6)		
	Female	86 (45.0)	187 (36.4)	4.39	.036
Marital status	Married	162 (84.8)	487 (94.7)		
	Unmarried	29 (15.2)	27 (5.3)	18.78	<.001
Educational level	Middle school or below	124 (64.9)	263 (51.2)		
	High school or above	67 (35.1)	251 (48.8)	10.64	.001
Household income (RMB)	≤5000	154 (80.6)	296 (57.6)		
	>5000	37 (19.4)	218 (42.4)	32.02	<.001
Location of residence	Urban	117 (61.3)	357 (69.5)		
	Rural	74 (38.7)	157 (30.5)	4.25	.039
Primary caregiver	Self‐care	104 (38.0)	334 (54.1)		
	Others	170 (62.0)	393 (12.8)	5.76	.016
Current work status	Employed	71 (37.2)	243 (47.3)		
	Unemployed	120 (62.8)	271 (52.7)	42.53	<.001
Current smoker	No	167 (87.4)	398 (77.4)		
	Yes	24 (12.6)	116 (22.6)	4.86	.028
Current drinker	No	180 (94.2)	614 (84.5)		
	Yes	11 (5.8)	113 (15.5)	11.00	.016
Regular physical activity	No	99 (51.8)	175 (34.0)		
	Yes	92 (48.2)	339 (66.0)	18.54	<.001
BMI (kg/m^2^)	<24	100 (52.4)	262 (51.0)		
	≥24	91 (47.6)	252 (49.0)	.11	.744
HbA1c (%)	≤7	62 (32.5)	156 (30.4)		
	>7	129 (67.5)	358 (69.6)	.29	.590
Duration of diabetes (year)	<10	67 (35.1)	255 (49.6)		
	≥10	124 (64.9)	259 (50.4)	11.85	<.001
Family history of diabetes	No	108 (56.5)	278 (54.1)		
	Yes	83 (43.5)	234 (45.9)	.71	.968
Stroke	No	154 (80.6)	465 (90.5)		
	Yes	37 (19.4)	49 (9.5)	12.59	<.001
Hypertension	No	50 (26.2)	202 (39.3)		
	Yes	141 (73.8)	312 (60.7)	10.44	.001
Coronary heart disease	No	129 (67.5)	418 (81.3)		
	Yes	62 (32.5)	96 (18.7)	15.22	<.001
Fatty liver	No	157 (82.2)	360 (70.0)		
	Yes	34 (17.8)	154 (30.0)	10.53	.001
Diabetic nephropathy	No	67 (35.1)	280 (54.5)		
	Yes	124 (31.4)	234 (50.0)	20.96	<.001
Diabetic retinopathy	No	97 (50.8)	337 (65.6)		
	Yes	94 (49.2)	177 (34.4)	12.58	<.001
Diabetic foot	No	167 (87.4)	480 (93.4)		
	Yes	24 (12.6)	34 (6.6)	6.53	.011

Abbreviations: BMI, body mass index; HbA1c, glycated hemoglobin A1c; MCI, mild cognitive impairment; RMB, renminbi.

### 3.3 Multivariate analysis of factors associated with MCI in the training set

The multivariate analysis of factors associated with MCI in the training set is shown in Table 3. Age (adjusted odds ratio (aOR) = 2.75, 95% confidence interval [CI]: 1.68–4.50, *p* < .001), marital status (aOR = 2.59, 95% CI: 1.36–4.93, *p* = .004), household income (aOR = .50, 95% CI: .30–.85, *p* = .010), regular physical activity (aOR = .59, 95% CI: .40–.87, *p* = .008), diabetic nephropathy (aOR = 1.68, 95% CI: 1.11–2.56, *p* = .015), and diabetic retinopathy (aOR = 1.55, 95% CI: 1.02–2.35, *p* = .042) were predictor variables independently associated with MCI by the multivariate logistic regression analysis.

**TABLE 3:** Multivariate analysis of factors associated with mild cognitive impairment (MCI) in the training set.

Variables	Category	*B*	*SE*	Adjusted OR (95% CI)	*p* Value
Age (year)	40–59 vs. ≥60	1.01	.25	2.75 (1.68–4.50)	<.001
Sex	Male vs. female	−.15	.22	.86 (.56–1.32)	.483
Marital status	Married vs. unmarried	.95	.33	2.59 (1.36–4.93)	.004
Educational level	Middle school or below vs. high school or above	.03	.24	1.03 (.65–1.64)	.901
Household income (RMB)	≤5000 vs. >5000	−.69	.27	.50 (.30–.85)	.010
Location of residence	Urban vs. rural	.27	.22	1.31 (.85–2.03)	.217
Primary caregiver	Self‐care vs. others	−.08	.20	.92 (.62–1.38)	.688
Current work status	Employed vs. unemployed	.39	.30	1.47 (.82–2.63)	.193
Current smoker	No vs. yes	−.31	.30	.74 (.41–1.34)	.316
Current drinker	No vs. yes	−.66	.39	.52 (.24–1.12)	.093
Regular physical activity	No vs. yes	−.53	.20	.59 (.40–.87)	.008
Duration of diabetes (year)	≥10 vs. <10	.14	.21	1.15 (.77–1.73)	.490
Stroke	No vs. yes	.50	.27	1.65 (.97–2.80)	.063
Hypertension	No vs. yes	.08	.23	1.09 (.69–1.70)	.721
Fatty liver	No vs. yes	.34	.22	1.41 (.91–2.18)	.129
Coronary heart disease	No vs. yes	−.38	.24	.68 (.43–1.10)	.117
Diabetic nephropathy	No vs. yes	.52	.21	1.68 (1.11–2.56)	.015
Diabetic retinopathy	No vs. yes	.44	.21	1.55 (1.02–2.35)	.042
Diabetic foot	No vs. yes	.46	.32	1.58 (.84–2.96)	.157

Abbreviations: CI, confidence interval; OR, odds ratio; RMB, renminbi; *SE*, standard error.

### 3.4 Development of the decision tree model

**FIGURE 1:**
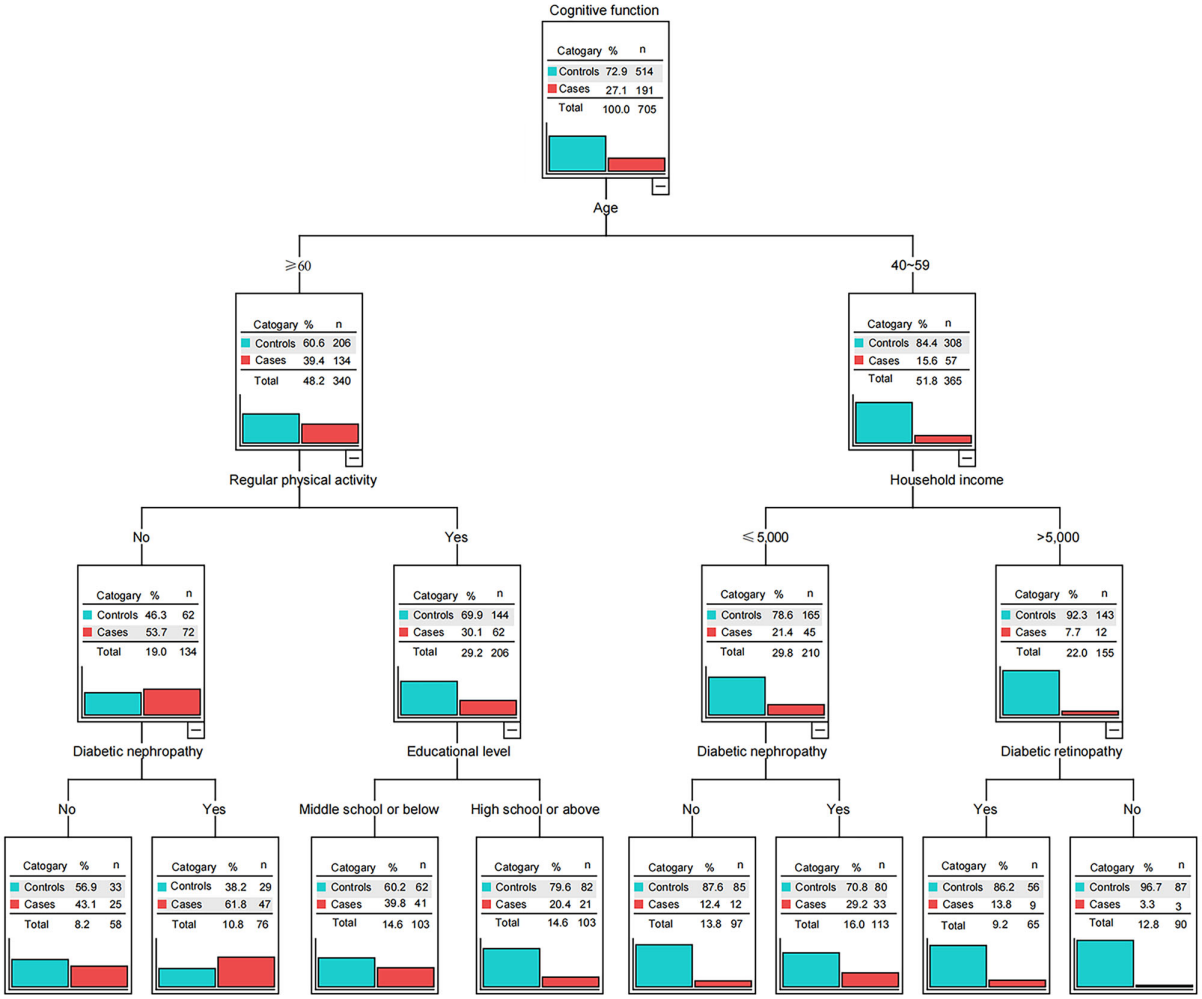
The established decision tree model.

A decision tree model was developed based on the 19 significant predictors variables (including age, sex, marital status, educational level, household income, location of residence, primary caregiver, current work status, current smoker, current drinker, regular physical activity, duration of diabetes, stroke, hypertension, coronary heart disease, fatty liver, diabetic nephropathy, diabetic retinopathy, and diabetic foot) found in the univariate analyses. According to the CHAID algorithm, age, educational level, household income, regular physical activity, diabetic nephropathy, and diabetic retinopathy were included in the decision tree model. The established decision tree model is shown in Figure 1. It had 15 nodes composed of 4 layers, and age is the most significant predictor variable.

### 3.5 Validation of the decision tree model

**FIGURE 2:**
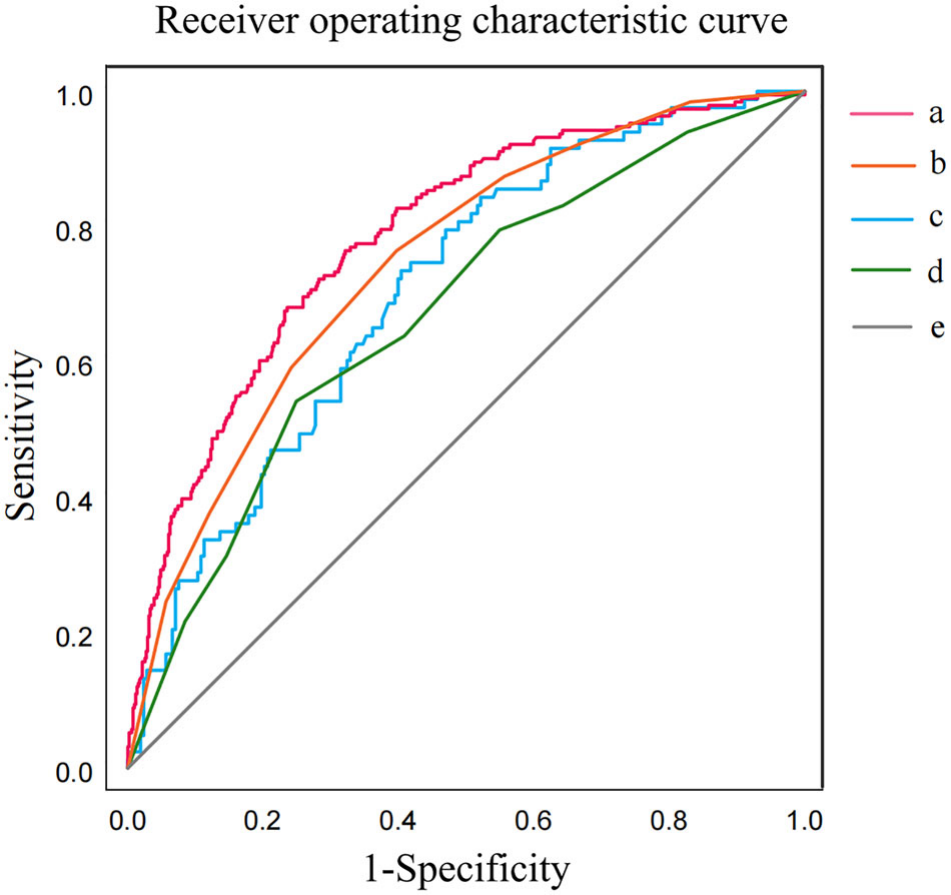
The receiver operating characteristic curves for the decision tree and multivariate logistic regression models: a—Multivariate logistic regression model in the training set; b—decision tree model in the training set; c—multivariate logistic regression model in the validation set; d—decision tree model in the validation set; e—reference line.

The ROC curves for the decision tree models and multivariate logistic regression models in the training and validation sets are shown in Figure 2. The AUC value of the decision tree models was .75 (95% CI: .71–.78) and .67 (95% CI: .61–.74) in the training and validation sets, respectively, and that of the multivariate logistic regression models was 0.76 (95% CI: .72–.80) and 0.69 (95% CI: .62–.75) in the training and validation sets, respectively.

## 4 DISCUSSION

This study developed a decision tree model to help identify MCI in patients with T2DM by comprehensively taking demographic characteristics, lifestyle factors, and T2DM‐related information into account. To the best of our knowledge, this was the first study attempting to use the CHAID decision tree analysis to identify MCI in patients with T2DM. The principle finding of this study was that compared to the multivariate logistic regression model, the established decision tree model had comparable predictive value and was well internally validated.

Previous studies that directly compared the performance of conventional logistic regression and decision tree models to solve prediction problems found comparable results. For example, Kuang et al. (2021) found that both logistic regression and decision tree models performed well at predicting the transition from MCI to Alzheimer's disease with ideal stability; Yu et al. (2024) found that the AUC value was .868 (95% CI: .821–.916) and .863 (95% CI: .814–.912) for the logistic regression and decision tree models to identify suicidal ideation in schizophrenia patients, respectively; and similar prediction accuracy in these two approaches was also observed in a study using the self‐reported clinical symptoms of dengue fever to predict potential dengue infection (Khosavanna et al., 2021). Compared to logistic regression, the main advantages of decision tree model are easy visualization and simplicity; the results are transformed to a set of decision rules that are similar to clinical reasoning; and there is an intuitive and straightforward explanation about how the decision process was made. This study added significantly to the existing body of knowledge by indicating that both logistic regression and decision tree models performed well at predicting MCI in patients with T2DM. Additionally, the decision tree model established in this study identified 6 key predictor variables with 15 nodes composed of 4 layers. It was simple and easy to understand without issues on multiple levels and nonrelevant splits. Therefore, the utilization of decision tree model to identify MCI in T2DM patients was more suggested in clinical practice considering its superior classification accuracy and simple presentation as well as interpretation of collected data.

In terms of the key predictor variables identified by the decision tree model, age, educational level, household income, and regular physical activity were well‐known factors that were frequently observed by previous work in the general population (Biessels et al., 2008; Dominguez et al., 2021; Jia et al., 2020; Zhang et al., 2019), whereas diabetic nephropathy and retinopathy were factors specifically limited to the diabetic population. As the most significant predictor variable for the identification of MCI in T2DM patients, the increased risk of MCI in those aged ≥60 observed in this study could be explained by the brain structure changes and decreased brain functioning that occur with increased age (Mankovsky et al., 2018). Educational level has been believed to be the strongest noncognitive factor affecting cognitive function (Fan et al., 2021; Pedraza et al., 2017), and the protective effects of high household income against MCI in T2DM may be attributed to higher social engagement and more social resources (Coughlin, 2020), as well as the ability to tolerate higher levels of neuropathology, which would be beneficial for the maintenance of cognitive function (Pais et al., 2021). Additionally, the role of exercise in maintaining cognitive function has been well documented (Donnelly et al., 2016; Houston et al., 2018), and this study contributed to the existing knowledge by supporting the protective effects of regular physical activity against MCI in patients with T2DM.

Diabetic nephropathy and retinopathy were the most prevalent microvascular complications of T2DM (Seewoodhary, 2021). Previous work has linked these two complications with a wide range of poor health‐related outcomes including reduced quality of life and increased risk of depression, anxiety, and bipolar disorder (Chen et al., 2023; Edalat‐Nejad et al., 2014; Khoo et al., 2019; Mahobia et al., 2021; Valluru et al., 2023). In addition to that, this study found diabetic nephropathy and retinopathy were both associated with higher risk of MCI. Potential mechanisms underlying the association between diabetic nephropathy and cognitive impairment included vascular dysfunction, lymphatic dysfunction, decreased clearance of uremic toxins, and hemodynamic changes during dialysis, which could lead to cognitive decline (Drew et al., 2019). Additionally, the impacts of diabetic retinopathy on cognitive function may be explained by the processes of shared pathways, including neuroinflammation and degeneration, vascular degeneration, and glial activation (Little et al., 2022). Therefore, the findings of this study strongly highlighted the importance of strengthening the management of diabetic complications including diabetic nephropathy and retinopathy for maintaining cognitive function of T2DM patients in clinical practice.

In conclusion, this study developed and validated a decision tree model to help identify MCI in T2DM patients in clinical practice by employing a large sample size. The established decision tree model based on age, educational level, household income, regular physical activity, diabetic nephropathy, and diabetic retinopathy performed well with comparable predictive value compared to the multivariate logistic regression model and was internally validated. However, some limitations need to be acknowledged. First, this study was hospital‐based. Therefore, whether the findings of this study can be generalized into those from community settings remains unclear. Second, though internally well validated, there was still a lack of external validation for the findings of this study. Third, this study only compared the performance of decision tree model with logistic regression, and it remains a possibility that other machine learning models, such as random forest, gradient boosting machine, and artificial neural network, could perform better than the decision tree model for the identification of MCI in T2DM patients. Therefore, future community‐based studies with comparisons between various machine learning models are warranted.

## AUTHOR CONTRIBUTIONS

**Rehanguli Maimaitituerxun**: Methodology; software; funding acquisition; formal analysis; visualization; writing—review and editing; writing—original draft. **Wenhang Chen**: Methodology; software; formal analysis; visualization; writing—review and editing; writing—original draft. **Jingsha Xiang**: Investigation; data curation; validation; writing—review and editing. **Yu Xie**: Data curation; validation; investigation; writing—review and editing. **Fang Xiao**: Data curation; validation; writing—review and editing; investigation. **Xin Yin Wu**: Data curation; validation; writing—review and editing; investigation. **Letao Chen**: Investigation; validation; writing—review and editing; data curation. **Jianzhou Yang**: Validation; writing—review and editing; methodology; supervision. **Aizhong Liu**: Validation; writing—review and editing; supervision; methodology. **Wenjie Dai**: Writing—review and editing; conceptualization; project administration; resources; methodology; funding acquisition.

## CONFLICT OF INTEREST STATEMENT

The authors have no conflicts of interest to declare.

### PEER REVIEW

The peer review history for this article is available at https://publons.com/publon/10.1002/brb3.3456.

## Data Availability

The data that support the findings of this study are available from the corresponding author, Wenjie Dai, upon reasonable request.
